# Effect of Thickness and Stitch Density on Low-Velocity Impact and Compression After Impact Properties of Stitched Composite Laminates

**DOI:** 10.3390/polym18070791

**Published:** 2026-03-25

**Authors:** Bangxiong Liu, Faliang Wang, Yina Zheng, Jiawen Huang, Shiyu Jiang, Wei Zhang

**Affiliations:** 1 School of Mechanical and Electronic Engineering, Jingdezhen University, Jingdezhen 333400, China; liubangxiong0798@126.com (B.L.);; 2 Jingdezhen Key Laboratory of Robotics for Intelligent Tea Harvesting, Jingdezhen University, Jingdezhen 333400, China; 3 Engineering Research Center of Railway Environment Vibration and Noise, East China Jiao Tong University, Nanchang 330013, China; 4 Jiangxi Province Key Laboratory of Precision Drive and Equipment, Jiangxi University of Water Resources and Electric Power, Nanchang 330099, China; 5 School of Mechatronics and Vehicle Engineering, East China Jiaotong University, Nanchang 330013, China

**Keywords:** stitched composite laminate, low-velocity impact, experimental study, delamination damage, compressive residual strength

## Abstract

In this work, experimental studies were conducted on the damage failure of laminated composite laminates under low-velocity impact and compressive failure behavior under compression after impact. The study primarily investigated the effects of stitch density, impact energy, and laminate thickness on the damage behavior of composite laminates. The experimental results indicate that at impact energies of 10 J, 15 J, and 20 J, the stitched specimens demonstrated higher impact resistance. When the stitch density was 10 × 10 mm, the average maximum impact force of the stitched specimens increased by 13.14%, 15.83%, and 21.48%, respectively, compared to the unstitched specimens. This was mainly attributed to the resin threads formed by the stitches, which enhance the through-thickness strength of the laminate, with the strengthening effect being positively correlated with stitch density. Under 20 J, the strength of the three groups of specimens with different stitching densities increased by 9.24%, 14.58%, and 21.48%, respectively, compared to the unstitched specimens. Under lower impact energies, the bending stiffness of the laminate itself was sufficient to resist the impact force, resulting in minimal differences in residual displacement among different specimens. Furthermore, the study found that under identical impact energy, stitch thread significantly suppressed delamination damage in thin specimens, whereas its effect on thick specimens was comparatively limited. The stitching also had a positive effect on the residual compressive strength of the specimens. Under 20 J impact energy, compared to the unstitched specimens, the residual compressive strength of the three groups of stitched specimens increased by 6.52%, 17.71%, and 27.48%, respectively. The mode of compression after impact failure also differed: unstitched laminated specimens mainly exhibited delamination damage, with cracks propagating along the width direction, while stitched laminated specimens demonstrated strength failure. Under axial compression, stress was released at the stitching points, leading to small-scale cracks along the fiber direction at these locations. Overall, the stitching process effectively enhances the impact resistance of laminated boards. Higher stitching density correlates with greater compressive residual strength, with this effect being more pronounced in thin-plate specimens.

## 1. Introduction

Composite laminates are widely used in industries such as aerospace, wind power, and rail transportation due to their high specific strength and stiffness, as well as excellent fatigue and corrosion resistance. Particularly in the aviation industry, they are often employed as the preferred engineering material [1,2,3]. During routine use and maintenance, composite materials are susceptible to external impacts, such as falling tools during aircraft inspections, runway gravel impacts, and hail strikes [4,5]. These impacts may leave no visible surface damage but can cause internal irreversible defects, such as matrix cracking and delamination [6,7]. Due to their invisible characteristics, such damages are easily overlooked. Once materials are compromised, structural rigidity and strength are significantly reduced, posing serious flight safety risks [8,9,10].

Owing to the inherent characteristics of laminated structures, composites exhibit relatively weak interlaminar properties and are prone to internal delamination, which severely shortens service life. Owing to the intrinsic characteristics of laminated structures, composite materials exhibit poor interlaminar properties and are prone to internal delamination, significantly reducing the service life of the structure. To enhance damage tolerance and inhibit delamination, researchers have explored approaches such as carbon fiber surface modification [11,12,13,14], interlayer reinforcement [15,16,17,18], and structural optimization [19,20,21,22,23]. Abdullah et al. [12] proposed that the geometry of helical multiwalled carbon nanotubes (HMWCNTs) helps lock the filler matrix in composites. Their tests on the impact resistance of composites containing 0.4% HMWCNTs showed that the HMWCNTs improved the low-velocity impact (LVI) and compression after impact (CAI) properties of the composite laminates. Ren et al. [13,16] modified both fibers and matrices, thoroughly discussing enhanced interfacial adhesion from the perspectives of physical entanglement and copolymerization. Experimental results demonstrated that surfaces modified with high-performance thermoplastic polymers could increase interfacial bonding strength and improve the performance of composite laminates. Wang et al. [19] employed a negative Poisson’s ratio structure to enhance the impact resistance of composite materials. Experimental results indicated that the fiber and matrix tensile damage in laminates with this structure could be reduced by approximately 40%. Inspired by natural biological structures, numerous scholars have mimicked these structures to enhance interlaminar properties, such as the wings of the Milvus migrans [20] and the scales of the coelacanth [21]. Olhan et al. [24,25] investigated the mechanical properties of woven (UD, 2D, 3D) composites fabricated using glass, basalt, and sisal fibers, exploring their potential applications in the automotive sector. Three-dimensional weaving processes [26] exhibit high complexity and shape limitations; Z-pin reinforcement techniques [27] can only enhance localized areas and are commonly used for preparing stiffener-reinforced laminates; nano-reinforcement [28] technologies face challenges in interface control and offer low cost-effectiveness. Constrained by the limitations of the aforementioned traditional interlayer reinforcement techniques, stitching technology [29,30,31] offers distinct advantages. By enhancing properties perpendicular to the plane of the laminate, it increases interlayer damage tolerance while significantly reducing manufacturing costs. This simple, direct, and effective process method also holds potential for large-scale production.

Numerous researchers have conducted a series of experiments to investigate the mechanical response and damage mechanisms of stitched composite laminates upon impact. Aymerich et al. [32,33,34] experimentally investigated the effect of stitching on the out-of-plane central impact performance of [0_3_/90_3_] s laminates. At higher impact energies, localized fiber breakage occurred; although stitching could not prevent delamination initiation or propagation, it reduced the delaminated area. When impact energy starts at 3 J, the damaged area of stitched specimens is reduced by an average of 22% compared to unstitched specimens. Byun et al. [35] and Francesconi et al. [36,37,38] observed that stitching restricts damage propagation both transversely and longitudinally, resulting in smaller damage areas compared to unstitched specimens. At an impact energy of 35 J, the damaged area decreased by approximately 30%. Lopresto et al. [39] noted that stitching effectively enhances damage tolerance in thicker laminates, while its effectiveness is relatively limited for thin laminates. Kim et al. [40,41] proposed a novel process to reduce carbon fiber bending and prevent fracture. A cylindrical stitch needle with embedded fibers was threaded through the preform. Pneumatic pressure was then applied to the cylinder, ejecting the stitch fibers from the needle to fabricate stitched T-stiffener laminate specimens. Although stitching suppresses delamination, it disrupts fiber continuity and can degrade mechanical properties [42,43,44,45], particularly tensile performance [46,47]. At a fiber density of 0.5%, the specimen’s strength decreased by 16.39%. Under the non-perforation impact loading, with the increase in impact energy, the reduction in delamination area of stitched composites with the large stitching space and the longitudinal pattern is not obvious. Due to the substantial manufacturing costs and extended production cycles associated with fabricating and preparing stitched composite laminates, coupled with the numerous factors influencing their impact and compression damage performance, existing experimental studies exhibit limitations [27,48,49,50]. Based on currently published literature, research on stitched composite laminates has predominantly focused on single parameters, such as: stitches per inch, stitching pattern, stitch density, etc. Lacking systematic understanding of the effects of stitch density and laminate thickness under low-velocity impact loading.

Therefore, this work first fabricated laminates using vacuum-assisted resin transfer molding (VARTM) combined with stitching. Specimens were cut to standard dimensions using a waterjet. LVI and CAI tests were conducted using a drop-weight impact tester and a universal testing machine. LVI damage was examined via ultrasonic C-scanning. The effects of impact energy, stitch density, and specimen thickness on impact damage and residual compressive strength were analyzed in detail. The study aims to reveal the damage mechanisms of stitched carbon fiber laminates under out-of-plane impact and clarify the failure modes in CAI.

## 2. Materials and Methods

### 2.1. Material Preparation

All specimens required for the experimental investigation were manufactured by our research group. The preparation process began with the stitching of preforms, followed by the manufacture of both stitched and unstitched carbon fiber laminates using the Vacuum Assisted Resin Transfer Molding (VARTM) process. The preforms were stitched, followed by the manufacture of both stitched and unstitched carbon fiber-reinforced polymer laminates via the VARTM process. The materials used were as follows: unidirectional carbon fiber fabric, model CF12-L300 (Zhongfu Shenying Carbon Fiber Co., Ltd., Lianyungang, China), with an areal density of 300 g/m^2^ and a thickness of 0.167 mm; The epoxy resin was R668 (Nan Ya Plastics, Taiwan, China). The detailed material parameters are shown in Table 1. The corresponding curing agent (brand H268) was sourced from BASF (BASF Group, Ludwigshafen, Germany). The epoxy resin exhibits low viscosity when mixed with the curing agent, offering excellent curing properties suitable for VARTM. The stitching thread was Kevlar-29 (1500 denier) supplied by DuPont (DuPont, Wilmington, DE, USA).

**Figure 1:**
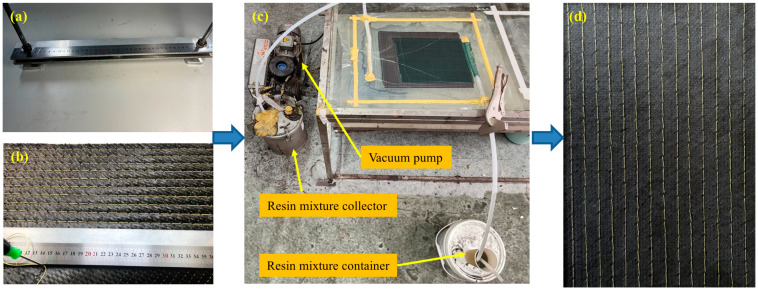
Process of Preparing Stitched Preforms, (**a**) auxiliary stitch device, (**b**) the stitching process of preforms, (**c**) VARTM process, (**d**) Stitched samples after processing.

After laying the unidirectional carbon fiber fabric according to the designed layup sequence, a modified lock stitch technique was employed. Since machine stitching is more applicable to wet layup processes like prepreg molding, it is less suitable for dry fabric processes. Using a stitching machine on dry carbon fiber fabric often causes hooks and pulls, resulting in poor stitching quality. Therefore, our group independently designed an auxiliary stitch device, as shown in Figure 1. Kevlar fiber was manually passed through the ply stack using this device to create a preform with a three-dimensional fiber architecture.

Following preform preparation, firstly, the laminates were fabricated via VARTM. During the molding process, the inlet port was clamped shut, and a vacuum pump was used to extract air from the vacuum bag. After maintaining the vacuum for a period, the outlet was also clamped to seal the system. Second, the curing agent and epoxy resin are mixed at a mass ratio of 1:5 at 50 °C. The prepared mixture is stored at atmospheric pressure in the resin cup. The vacuum pump then drew the mixture into the fiber preform inside the bag. Once the resin had thoroughly infiltrated the preform, the excess mixture flowed into a resin collector. Finally, the laminate was cured under vacuum at room temperature for 24 h before being demolded.

**Figure 2:**
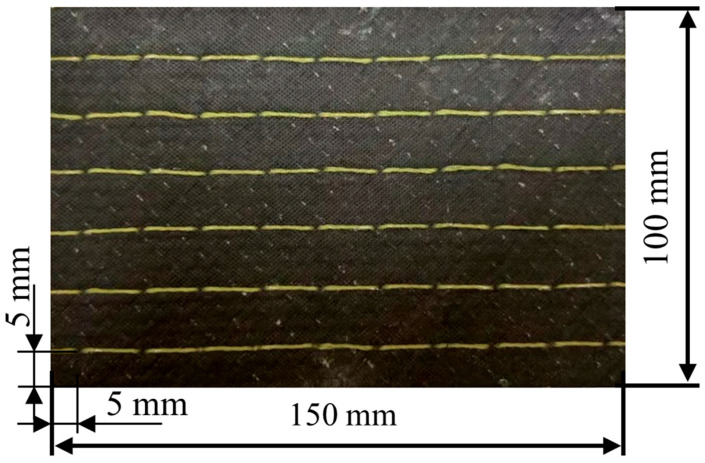
Stitched specimens for low-velocity impact testing.

The laminates were cut into standard impact specimens using a CNC waterjet cutter. The specimens have a geometry of 150 × 100 mm and a fiber volume fraction of 52%. To ensure consistency among the stitched samples, the first row of stitches was placed 5 mm from the specimen edge, as illustrated in Figure 2. To investigate the effects of different thicknesses and stitching densities on the LVI behavior of stitched carbon fiber composites, the impact specimens were prepared with layup sequences [45/0/−45/90] _s_ and [45/0/−45/90] _2 s_, with a single-layer thickness of 0.3 mm. The specimens with varying stitch densities and thicknesses are shown in Table 2. Specimens designated with 0 J impact energy were non-impacted and served as controls for the compression-after-impact tests. Three replicates were prepared for each parameter combination at each impact energy level (including 0 J), resulting in a total of 96 specimens.

### 2.2. LVI Test

**Figure 3:**
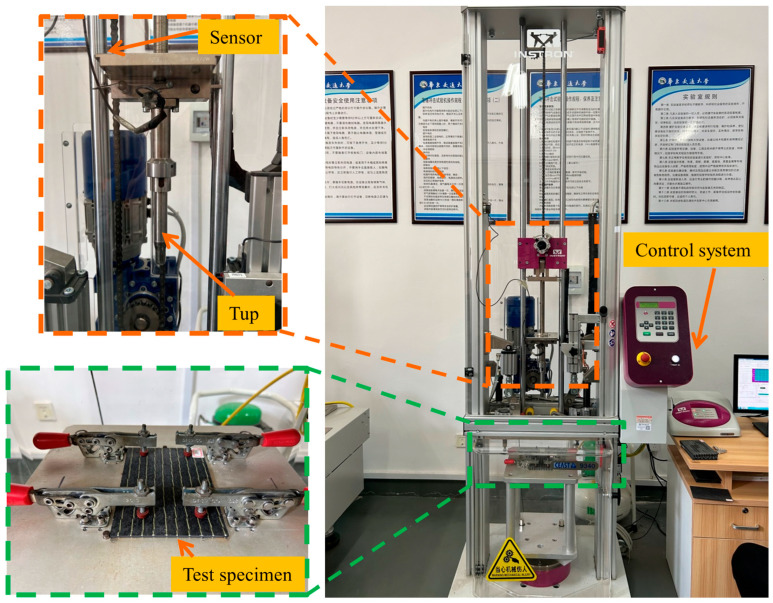
Instron CEAST 9340 drop weight impact tester.

LVI tests were conducted on the six specimen groups from the previous section using a drop-weight impact testing machine (Instron CEAST 9340, Instron Corporation, Norwood, MA, USA), as illustrated in Figure 3. A hemispherical steel impactor with a 16 mm diameter was employed, and the total system mass was 5.3 kg. The device incorporates a secondary impact prevention mechanism to ensure that specimens undergo only a single impact. The testing process strictly followed the ASTM D7136/D7136M standard [51] for drop weight impact testing. Impact energy levels of 10 J, 15 J, and 20 J were set, with three repeated tests conducted at each level. The drop height of the punch was calculated based on the preset impact energy using the formula *E* = *mgh*, where *E* is the impact energy, *m* is the mass of the tup, and *g* is the gravitational acceleration (9.81 m/s^2^). The tup reaches its maximum velocity upon contact with the laminate, which can be calculated using the formula *E* = 1/2*mv*^2^. During the impact process, the displacement, force, and velocity of the impactor were simultaneously recorded by a data acquisition system integrated into the measurement apparatus.

### 2.3. CAI Test

Following the impact tests, the stitched and unstitched specimens contained induced damage. To obtain the residual compressive strength of the specimens, compression tests were conducted on the computer-controlled electronic universal testing machine ETM 105D (Wance Technologies Ltd., Shenzhen, China). The machine has a load capacity ranging from 0.4 to 100 kN, a displacement resolution of 0.025 μm, and a crosshead speed adjustable from 0.001 to 500 mm/min, meeting the test requirements.

**Figure 4:**
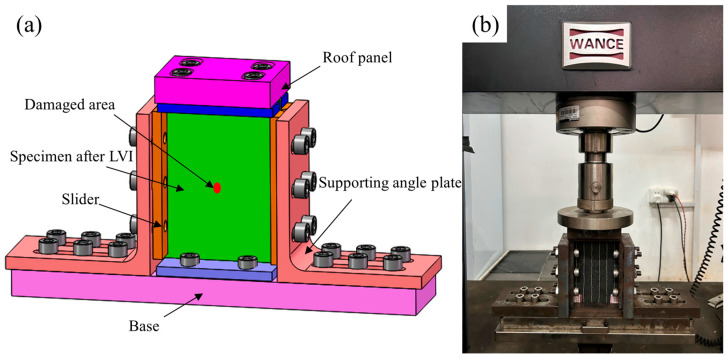
The damaged specimen was compressed, (**a**) CAD model of the compression support fixture, (**b**) physical model of the compression support fixture.

The test strictly followed the compression residual strength standard ASTM D7137 [52]. The fixtures used were custom manufactured according to this standard, as shown in Figure 4. The CAD model of the compression support fixture is presented in Figure 4a. The fixture consists of a base, a roof panel, support angle plates, and sliders. The top plate assembly comprises two sliders and the roof panel connected via bolts. After placing the specimen into the fixture, its upper edge surface contacts the top assembly. The positions of the left and right sliders were adjusted to ensure the specimen was centered within the fixture and the blocks were then securely tightened with bolts. A specific preload was applied during tightening to prevent specimen slippage during compression. The base of the fixture was bolted to the compression testing machine and a photograph of the manufactured fixture is shown in Figure 4b. The loading rate in the compression test was set at 1.25 mm/min. Observing the load–displacement curve, when the load dropped to approximately 70% of the peak load, it indicated that compression failure had occurred. This indicates that the compression test has concluded.

### 2.4. Detection Equipment

**Figure 5:**
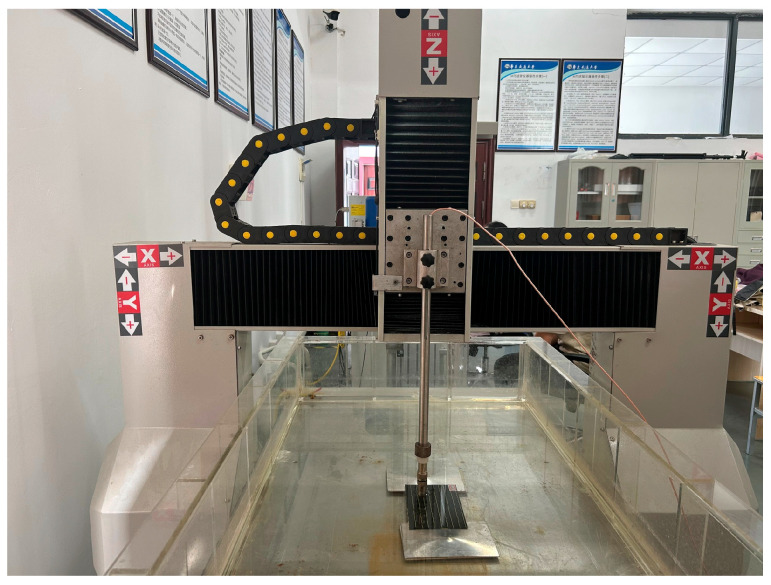
Water-immersed ultrasonic C-scan device UTScanMaster-6090.

Nondestructive inspection of the damaged specimens was performed using the water-immersed ultrasonic C-scan device UTScanMaster-6090 (Nanchang Hangkong University, Nanchang, China), as shown in Figure 5. This device has a maximum inspection range of 500 × 500 × 10 mm, a scanning resolution of 0.1 mm, and a transducer frequency of 5 MHz. Given the relatively minor damage, a 50 × 50 mm area centered on the impact zone was scanned for all specimens to improve inspection efficiency. The scanning speed was set to 50 mm/s.

## 3. LVI Results and Discussion

### 3.1. Impact Responses of Unstitched and Stitched Laminates

**Figure 6:**
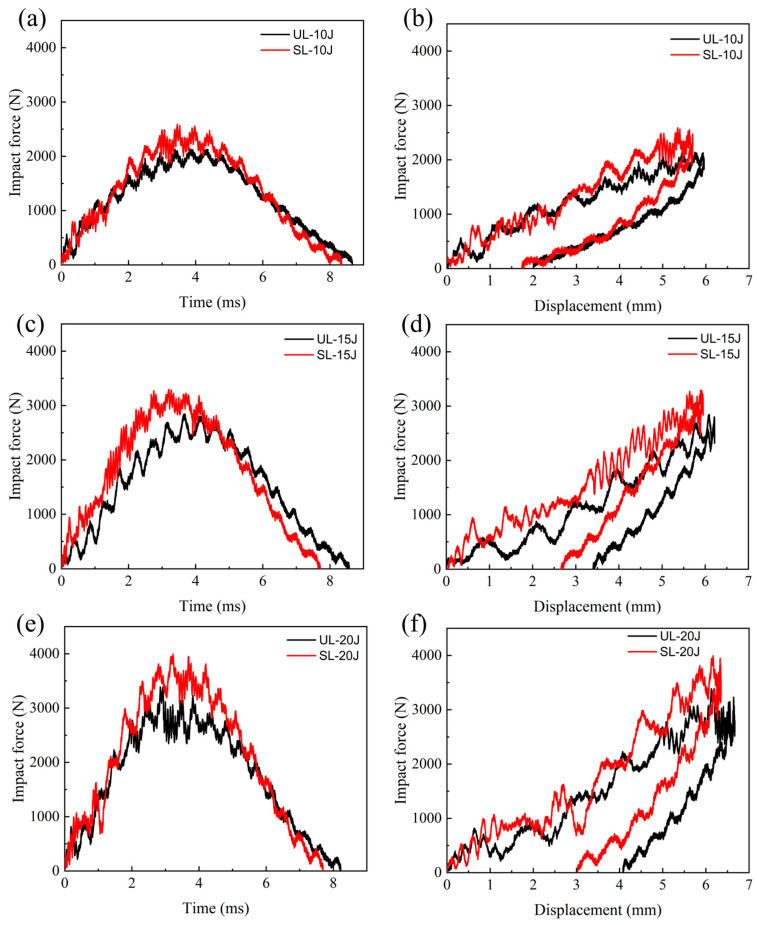
Mechanical response of unstitched specimens and stitched specimens with 10 × 10 mm stitch density under LVI at different energies; impact force-time curves of (**a**) 10 J, (**c**) 15 J, (**e**) 20 J; impact force–displacement curves of (**b**) 10 J, (**d**) 15 J, (**f**) 20 J.

The mechanical responses of unstitched and stitched specimens under different impact energies are shown in Figure 6, representing the impact force-time and impact force–displacement curves of unstitched specimens and specimens with a stitch density of 10 × 10 mm under three impact energies of 10 J, 15 J, and 20 J, respectively. As observed in Figure 6a,c,e, the impact force-time curves of both specimens exhibit similar characteristics. The impact force increases continuously throughout the impact process, with the curve showing an upward oscillating at the initial stage. This primarily arises from the elastic vibration generated during the initial contact between the laminate and the impact tup bottom. A brief initial drop in force occurs shortly after contact, attributable to interfacial debonding between fibers and the matrix due to the relatively low matrix strength. Additionally, the relatively stable amplitude in the early to middle stages of the curve indicates that damage has begun accumulating within the material. As the impact force approaches its peak, significant damage has already developed within the laminate, causing a degradation in bending stiffness. The curve tends to violent oscillations. Due to the continuous degradation of the overall stiffness of the composite material during the impact process, part of the impact energy is dissipated. The increasing trend in the impact force gradually slows. Once the impact force reaches the peak force, the impactor velocity drops to zero, and rebound begins; the impact force then decreases until it reaches zero as the impactor completely separates from the laminate surface.

Comparison of impact forces reveals that, for the same impact energy, the peak force of stitched specimens is higher than that of unstitched specimens. This is because the stitched specimen forms stitch resin columns by integrating implanted stitches with resin curing, enabling it to absorb part of the impact load and thereby increasing the peak impact force. Moreover, the impact force-time curve indicates that the stitching effect improves with increasing impact energy. Among the tested impact energy levels, higher energy levels demonstrated greater stitching effectiveness. The impact force–displacement relationships are shown in Figure 6b,d,f. Residual displacement persists after the impactor leaves the material surface. Experimental observations indicate that this residual displacement is not caused by the indentation damage resulting from the impact. This may be attributed to the laminate plate remaining bent when the impactor departs, having not yet fully recovered to a near-zero stable state. This is consistent with the conclusions of the simulation analysis by Zhou et al. [53]. Under the same impact energy, both the maximum displacement and the residual displacement of stitched specimens are smaller than those of unstitched specimens. Since the stitch–resin columns are aligned parallel to the impact direction, they enhance the through-thickness strength and improve the impact resistance of the stitched structure.

**Figure 7:**
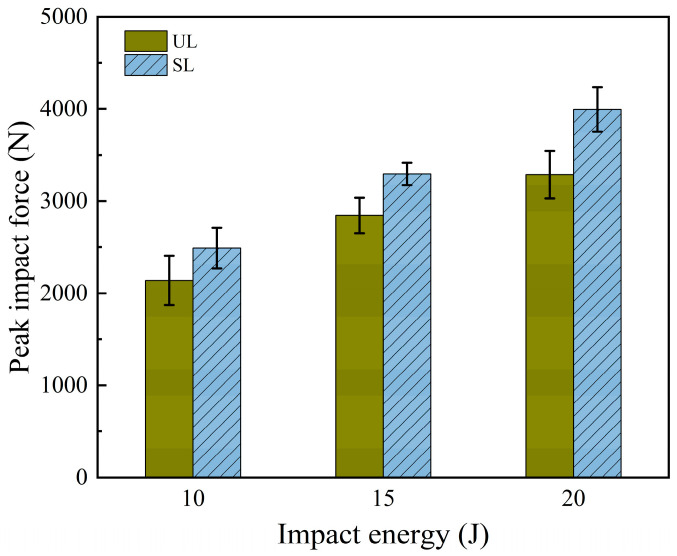
Average peak impact forces of unstitched specimens and stitched specimens with 10 × 10 mm stitch density.

The relationship between different impact energies and peak impact forces is shown in Figure 7. It can be observed that at impact energies of 10 J, 15 J, and 20 J, the average peak impact force of stitched laminates increased by 13.14%, 15.83%, and 21.48%, respectively, compared with unstitched laminates. This further confirms that the stitching process enhances the impact performance of laminates, and the improvement becomes more significant with increasing impact energy.

**Figure 8:**
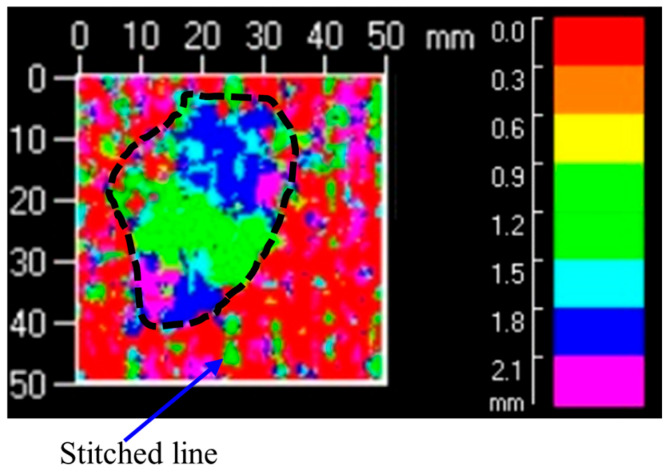
Ultrasonic C-scan results of the impact specimen with a stitch density of 10 × 10 mm.

After visual inspection, the damaged area of the specimens was examined using an ultrasonic C scan device. The scanning region was a central 50 × 50 mm area. Figure 8 shows a typical projection of delamination damage in a stitched specimen. Since the single-layer thickness of the specimen was 0.3 mm, the scanning step spacing was set to 0.3 mm. The color scale on the right side of the image provides a qualitative representation of delamination damage. Different colors indicate the location and delamination damage. Higher values on the scale correspond to damage farther from the scanning probe. Figure 8 shows that the specimen surface is relatively rough, with large areas of red and purple. The red indicates the region near the probe, representing the impact front of the specimen. In contrast, the purple indicates the region away from the probe, representing the impact back of the specimen. The stitch lines on the specimen surface primarily appear as red or purple striped areas in the ultrasonic C-scan image. The delaminated damage is outlined by the black dashed area in the image, exhibiting an elliptical morphology predominantly green in color. The delaminated damage in the LVI specimens was primarily distributed in the middle layer and gradually extended toward the back side, which is consistent with the findings from the visual inspection.

**Figure 9:**
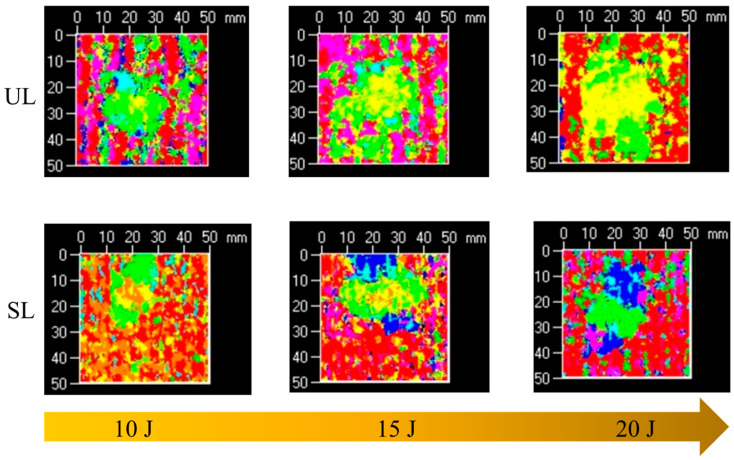
Ultrasonic C-scan images of unstitched and stitched specimens with 10 × 10 mm stitch density under different impact energies.

Ultrasonic C-scan imaging of unstitched and stitched specimens under different impact energies is shown in Figure 9. Damage projections reveal that both unstitched and stitched specimens exhibit similar elliptical features for out-of-plane impact damage. Delamination propagates outward from the central region, exhibiting an irregular distribution pattern. The long axis of the delamination is oriented along the fiber direction of the adjacent lower ply, with the most severe damage occurring on the side opposite to the impact surface. At the same impact energy, the delamination damage in stitched specimens is smaller than that in unstitched specimens, indicating that stitches can reduce the delamination area and suppress delamination damage. The damaged area is directly proportional to the impact energy, reaching its maximum at 20 J. The rate of delamination increase in unstitched specimens is higher than in stitched specimens, demonstrating that the inhibitory effect of stitching on delamination becomes more pronounced at higher impact energies. These observations further confirm that, in addition to enhancing the through-thickness strength and dissipating impact energy, the stitches also provide bridging forces in the thickness direction, effectively restraining delamination expansion.

### 3.2. Effect of Stitch Density on Impact Results

The previous section analyzed and discussed how the stitching process can effectively enhance the LVI performance of laminates and suppress delamination damage, with the effect becoming more pronounced at higher impact energies. This section focuses on the influence of stitching density on impact performance.

**Figure 10:**
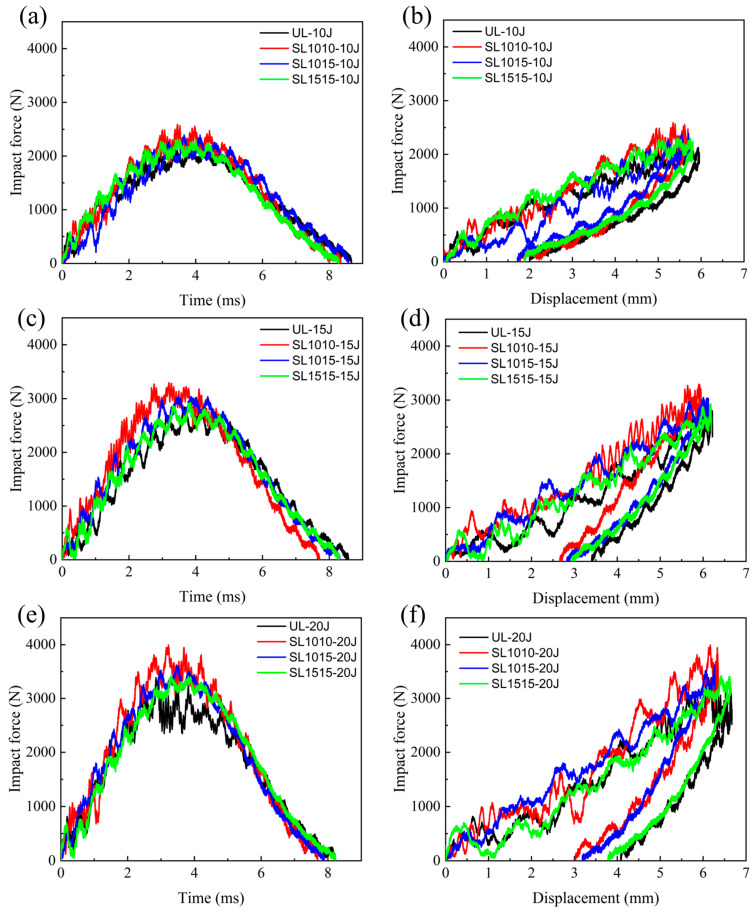
Mechanical response of group UL, group SL1010, group SL1015, group SL1515 under LVI at different energies; impact force-time curves of (**a**) 10 J, (**c**) 15 J, (**e**) 20 J; impact force–displacement curves of (**b**) 10 J, (**d**) 15 J, (**f**) 20 J.

Figure 10 shows the mechanical response curves of four specimen groups (UL, SL1010, SL1015, SL1515) under different impact energies. The impact force-time curves in Figure 10a,c,e reveal a consistent trend across all specimens and energy levels: an initial rise in impact force, followed by a peak at which the impactor velocity drops to zero, and rebound begins, after which the force declines until it reaches zero as the impactor separates from the laminate surface. At an impact energy of 10 J, the peak impact force of the stitched specimens exceeds that of the unstitched specimens. The peak force values for the groups SL1010, SL1015, and SL1515 were relatively close. When the impact energy increased to 20 J, the impact forces among these groups showed significant differences, with the group SL1010 exhibiting the highest impact force. This indicates that stitching enhances the impact resistance of laminates. Higher stitching density increases the number of stitches in the impact zone, and the resin columns formed by these stitches collectively contribute to resisting impact energy. Therefore, greater stitching density yields a more pronounced improvement in the impact performance of the laminate.

The impact force–displacement curves under 10 J, 15 J, and 20 J impact energies are presented in Figure 10b,d,f. The residual displacements among different specimens differ only slightly by under 10 J. At 20 J, however, the residual displacement of the group SL1010 is 3.0 mm, compared with 4.1 mm for the group UL. This is due to the fact that while stitching can enhance the bending stiffness of laminated boards, at lower impact energies, the inherent bending stiffness of the laminated board itself is sufficient to resist the impact energy, and the improvement from stitching is not significant. When impact energy increases, the bending stiffness of the laminate itself is insufficient to resist the impact energy. The addition of stitching enhances its bending stiffness, with the reinforcement effect being directly proportional to the stitching density.

**Figure 11:**
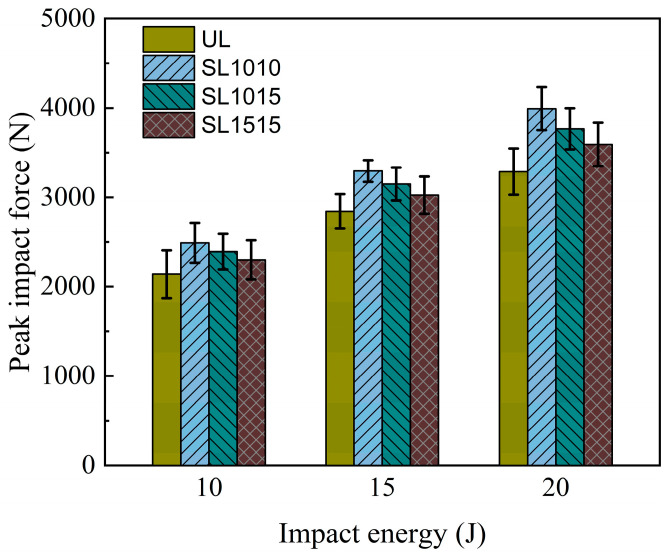
Average peak impact forces under different impact energies for the groups UL, SL1010, SL1015, and SL1515.

The average peak load for the four groups (UL, SL1010, SL1015, SL1515) under different impact energies is shown in Figure 11. Under the impact energy of 10 J, the peak loads of the stitched groups SL1010, SL1015, and SL1515 increased by 13.14%, 11.89%, and 7.57%, respectively. When the impact energy was 15 J, the increases were 15.83%, 10.72%, and 6.34%. At 20 J, the corresponding improvements reached 21.48%, 14.58%, and 9.24%. These data indicate that the stitching enhancement effect is related to both impact energy and stitch density: higher stitch density together with greater impact energy leads to a more pronounced improvement in performance.

**Figure 12:**
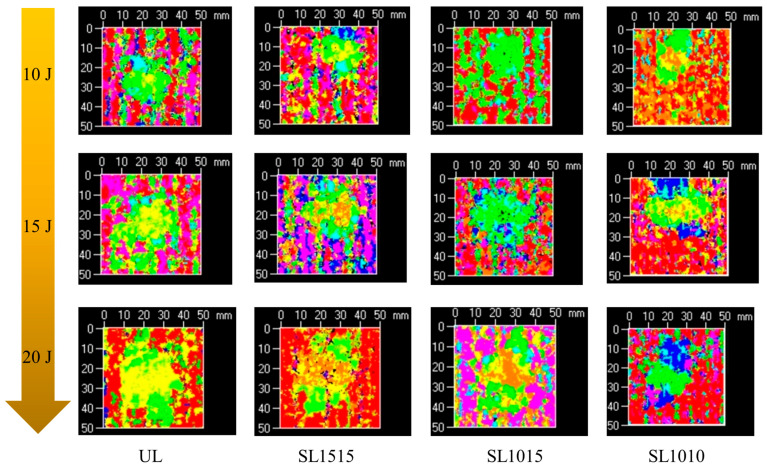
Ultrasonic C-scan images of groups UL, SL1515, SL1015 and SL1010 subjected to impact energies of 10 J, 15 J, and 20 J.

Figure 12 displays ultrasonic C scan images of specimens with different stitching densities under various impact energies. The damage projections reveal that out-of-plane impact leads to similarly shaped elliptical delamination damage across specimens with varying stitch densities. Under an impact energy of 10 J, the delamination damage areas of specimens with different stitching densities and of unstitched specimens showed only minor differences, indicating that stitching cannot prevent the initiation of delamination. However, as impact energy increases, the effects of stitching density on delamination damage become more pronounced, consistent with the findings of Tan et al. [30]. With increasing impact energy, the difference in delamination size caused by stitch density becomes more noticeable. At 20 J, it can be clearly observed that the specimen group with the highest stitch density (SL1010) exhibits the smallest delamination area. Although stitching does not prevent delamination, it is a simple and effective interfacial reinforcement method that provides bridging forces in the thickness direction of the laminate. As stitch density increases, the number of stitches participating in bridging within the impact region increases correspondingly, thereby more effectively restraining the propagation of delamination damage.

### 3.3. Effect of Plate Thickness on Impact Results

**Figure 13:**
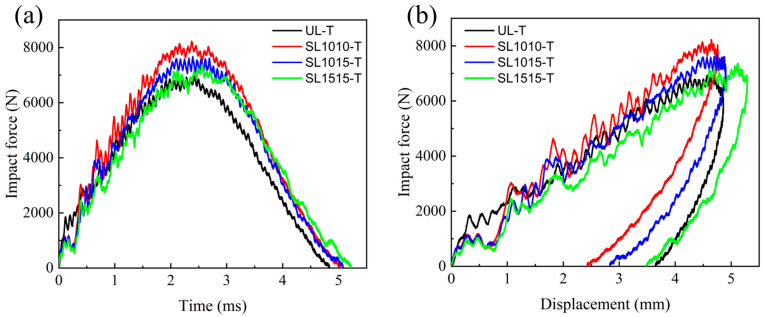
Mechanical response of groups UL-T, SL1010-T, SL1015-T, SL1515-T with a thickness of 4.8 mm under 20 J impact energy, (**a**) impact force-time curves, (**b**) impact force–displacement curves.

To better understand the effect of thickness on the LVI behavior of stitched specimens, impact tests were conducted on specimens with a thickness of 4.8 mm and different stitching densities. Detailed discussions were carried out regarding impact force, displacement, and delamination damage. Figure 13 shows the mechanical response of specimens with varying stitch densities at a thickness of 4.8 mm under 20 J. Comparing the impact force-time curves in Figure 13a and Figure 10e, specimens of different thicknesses exhibit similar overall trends. In the initial stage of impact, the curves show pronounced oscillations, indicating that damage has initiated and is propagating rapidly within the laminate. Continued oscillations near the peak load further suggest substantial internal damage and progressive stiffness degradation. Notably, the oscillations near the peak are less pronounced in thicker stitched specimens compared with thinner ones, which can be attributed to the greater bending resistance and lower deflection of the thicker laminates. Moreover, thickness also significantly affects impact duration; as specimen thickness increases, the impact contact time gradually decreases.

Comparing the impact force–displacement curves in Figure 13b and Figure 10f, the maximum displacement of group SL1010-T is 4.77 mm with a residual displacement of 2.43 mm. Group SL1010 shows a maximum displacement of 6.35 mm and a residual displacement of 3.02 mm. Both maximum and residual displacements decrease as the laminate thickness increases, which can be attributed to the greater stiffness of thicker plates in resisting deformation. Under the impact energy of 20 J, the peak load of the stitched specimen groups SL1010-T, SL1015-T, and SL1515-T increased by 18.42%, 10.48%, and 5.99%, respectively. It is evident that higher stitching density enables greater resistance to impact energy, while the improvement for thin plates reached 21.48%, all falling within the range reported for similar material systems [45]. Notably, our results indicate that stitching density has a relatively minor effect on enhancing the impact resistance of thick plates, suggesting that the improvement in impact resistance of laminated boards through stitching density exhibits thickness dependency, a finding not previously reported in the current literature.

**Figure 14:**
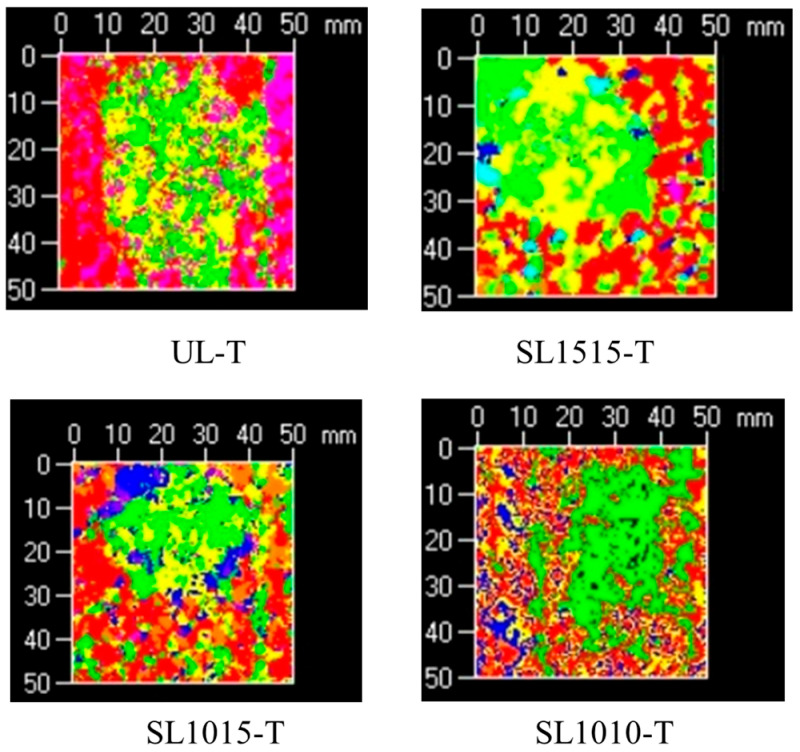
Ultrasonic C-scan images of four groups of specimens (UL-T, SL1010-T, SL1015-T, SL1515-T) with a 4.8 mm thickness under 20 J impact energy.

Figure 14 presents the Ultrasonic C scan results of specimens with a thickness of 4.8 mm and different stitching densities under 20 J impact energy. Comparing the damage projections of the four groups (UP-T, SL1010-T, SL1015-T, SL1515-T), it can be observed that the damage morphology tends to exhibit irregular vertical stripes. The difference in delamination area between stitched and unstitched specimens is relatively small, indicating that the inhibitory effect of stitching on delamination is not pronounced in these thicker specimens. This is likely due to the higher inherent stiffness of the thick laminates, which may have limited the reinforcing contribution of the stitches under the impact energy levels tested in this study. Additionally, compared to thin plates, thick plates exhibit a larger area of delamination. This is because as the laminate thickness increases, the stress experienced by each layer also increases. Consequently, the stress differential between adjacent upper and lower layers widens, leading to a proportional increase in the damaged area. Therefore, under the same impact energy, thin plates primarily dissipate impact energy through matrix damage, while thick plates do so through delamination. Zhou et al. [54] observed a similar trend for composite materials subjected to single and multiple impacts.

## 4. CAI Results and Discussion

### 4.1. CAI Strength

**Figure 15:**
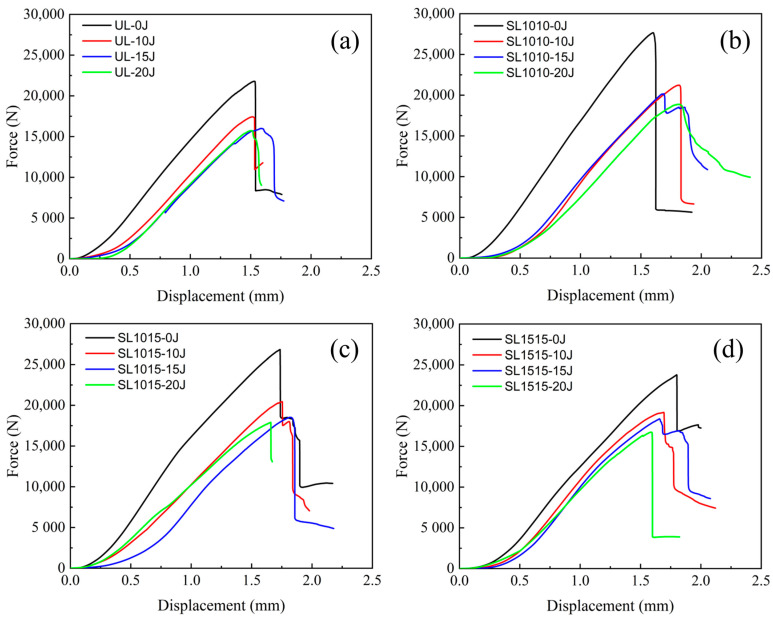
CAI response curves for 2.4 mm thick specimens under impact energies of 0 J, 10 J, 15 J, 20 J, (**a**) group UL, (**b**) group SL1010, (**c**) group SL1015, (**d**) group SL1515.

The compressive response of CAI in unstitched and differently stitched thin plate specimens is shown in Figure 15, where 0 J indicates specimens without impact damage. The load–displacement curves generally increase at the beginning of the compression test until they reach the brittle fracture load of the laminate, after which they drop abruptly. At this moment, the specimen emits a clear fracture sound during the test. As can be seen from the figure, the undamaged specimens exhibit higher compressive strength and greater stiffness than the damaged specimens. This is because during the CAI damage process, impact damage induces crack initiation and propagation, significantly reducing the residual compressive strength of the structure. Within the displacement range of 0.0–0.5 mm, the curves of the impacted specimens rise relatively slowly. When the displacement exceeds 0.5 mm, the rate of increase changes: the slope increases, then remains linear until the fracture load is reached.

**Figure 16:**
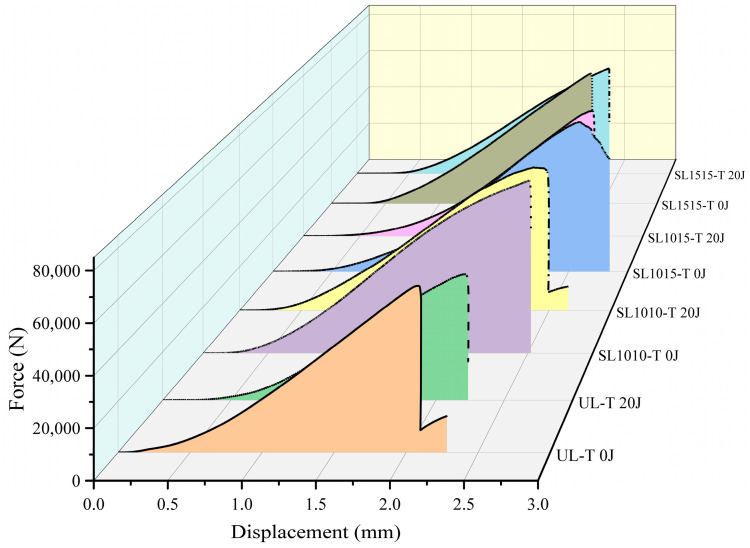
CAI response curves for 4.8 mm thick specimens under 20 J.

When the impacted specimens are subjected to higher impact energy, their fracture load is lower. This is attributed to the fact that higher impact energy induces more extensive delamination, thereby compromising the structural compressive strength. Figure 16 presents the CAI compression response of different specimens with a thickness of 4.8 mm under an impact energy of 20 J. For specimens with varying stitch densities, the fracture load values are all higher than that of the unstitched specimen. Furthermore, as the stitch density increases, the fracture load also rises.

The residual compressive strength of CAI can intuitively represent the strength of damaged specimens after impact. According to test standard ASTM D7137 [52], the formula [25] for calculating the residual compressive strength of CAI is as follows:

(1) $$\sigma_{\mathrm{CAI}}=\frac{F_{\max}}{w\times t}$$

where *σ*_CAI_ denotes the ultimate compressive residual strength, MPa; *F*_max_ denotes the maximum compressive force before failure, N; *w* denotes the specimen width, mm; *t* denotes the specimen thickness, mm.

**Figure 17:**
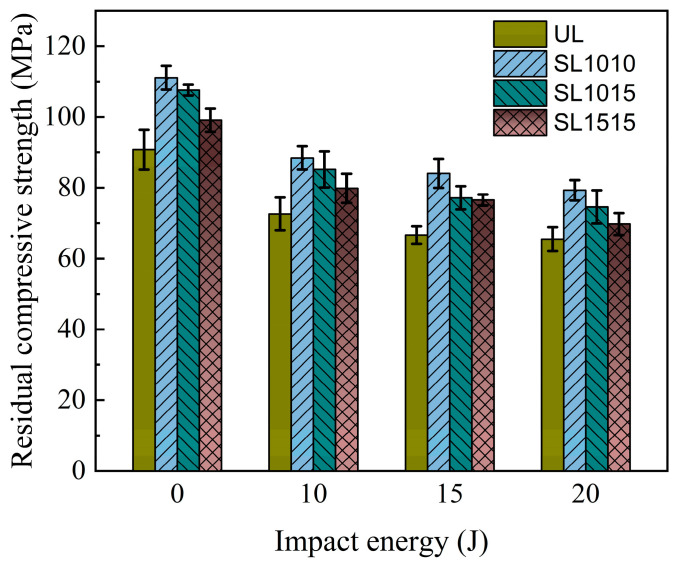
Residual compressive strength of CAI in groups UL, SL1010, SL1015, SL1515 with thickness of 2.4 mm under 0 J, 10 J, 15 J, and 20 J.

**Figure 18:**
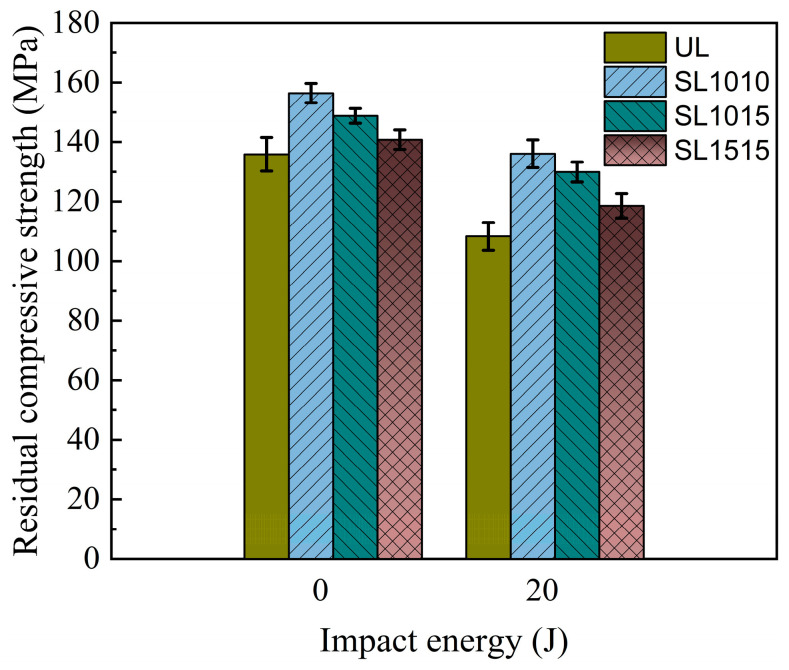
Residual compressive strength of CAI in groups UL, SL1010, SL1015, SL1515 with thickness of 4.8 mm under 0 J, 10 J, 15 J, and 20 J.

Figure 17 shows the residual compressive strength of the 2.4 mm specimens under different impact energies. As can be seen from the figure, the strength of all specimens decreases with increasing impact energy. At the same impact energy, the residual compressive strength of stitched specimens is consistently higher than that of unstitched specimens. At an impact energy of 10 J, the residual strength values of the groups SL1515, SL1015, and SL1010 increased by 9.19%, 18.56%, and 22.39%, respectively, compared to the UL specimens. When the impact energy was increased to 20 J, the strength values of the sutured specimens increased by 6.52%, 17.71%, and 27.48%, respectively. These results indicate that stitching enhances compressive residual strength of CAI after impact, with greater improvement observed at higher stitch densities. The residual strength is closely related to the extent of delamination caused by impact damage. When densely packed stitching collectively resists impact energy, it reduces delamination damage in the laminate. Therefore, higher stitching densities lead to greater compressive residual strength. The residual strength of the thick plate under 20 J impact energy is shown in Figure 18. Compared with Figure 17, it is evident that under 20 J impact, the thin plate experiences a faster decline in residual compressive strength than the thick specimen. This is primarily because the thick plate exhibits greater bending resistance, resulting in superior impact resistance performance.

### 4.2. Failure Mode

**Figure 19:**
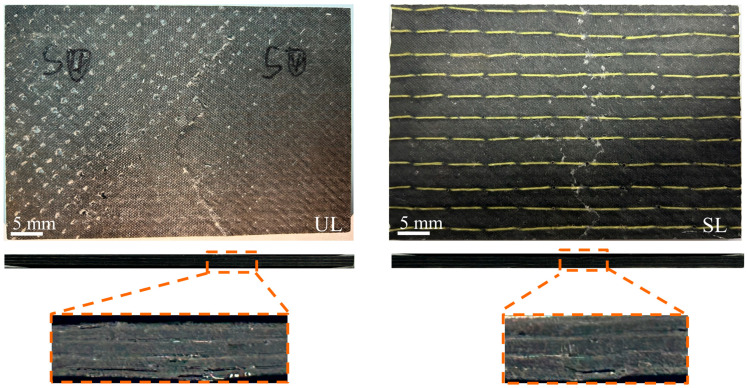
Typical failure modes of unstitched and stitched specimens after CAI.

Compression tests were conducted on all specimens, using unimpacted specimens as reference samples. The test concluded when a fracture explosion sound was heard, indicating specimen failure. Typical failure modes for unstitched and stitched specimens after CAI are shown in Figure 19.

From the front view, the compressed specimens exhibit cracks perpendicular to their length direction in the central region, spanning the entire specimen width. It is also evident that the cracks in unstitched specimens are broader than those in stitched ones. This is primarily due to stress concentration in the impact damaged area after axial compression. For the stitched specimen, the stress is redistributed along the stitch lines. Therefore, cracks in the stitched specimens propagate outward from the stitching points under compression, whereas cracks in the unstitched specimens propagate inward along the internal structure.

The top view further reveals clear delamination damage along the sides of the unstitched specimen, which has extended to the specimen edges. In the stitched specimen, however, delamination is confined and has not reached the edges. This indicates that the stitching effectively hinders crack propagation and reduces delamination damage. It can be concluded that the failure mode of the unstitched specimen is primarily interlaminar propagation, while the failure mode of the stitched specimen is fracture-strength failure.

## 5. Conclusions

This work employed the VARTM process to manufacture test specimens with varying thicknesses and stitching densities required for experimentation. LVI tests and CAI tests were conducted on these specimens. To investigate the LVI damage mechanisms and compressive failure modes of the stitched specimens, interlaminar damage was characterized via ultrasonic non-destructive testing. The effects of stitching density, specimen thickness, impact energy, and other relevant parameters on the failure mechanisms were analyzed, leading to the following conclusions:

(1) The through-thickness reinforcement provided by the stitched resin columns enhances the structural impact resistance, resulting in higher peak contact forces for stitched specimens. This improvement becomes more pronounced with increased stitch density. At 20 J, the average maximum impact force of specimens with a stitch density of 15 × 15 mm increased by 9.24% compared to unstitched specimens. When the stitch density was changed to 10 × 10 mm, the improvement rose to 21.48%. Furthermore, both the maximum and residual displacements of stitched specimens are lower than those of unstitched ones, with displacement being inversely proportional to stitch density. At an impact energy of 10 J, the displacement difference between specimens is minimal. As the energy increases to 20 J, this difference grows significantly, measuring 1.1 mm. This is because, although stitching can enhance the bending stiffness of laminated panels, when impact energy is low, the inherent bending of the laminated plate itself is sufficient to resist the impact energy, and the improvement from stitching is not significant at low energy levels.
(2) Damage to specimens after impact primarily consists of matrix damage and delamination damage. At different impact energies, delamination damage in stitched specimens is less than in unstitched specimens. Stitching cannot prevent delamination from occurring, but it can suppress its propagation, with stitch density inversely proportional to the delamination area. Furthermore, at the same impact energy, stitching improves delamination in thin plates more effectively than in thick plates.
(3) The dominant failure mode for unstitched specimens under CAI is delamination-dominated failure, with cracks propagating along the width direction. In stitched specimens, stress concentrations induced by axial compression are relieved along the stitch rows, generating small cracks aligned with the fiber direction at the stitch locations. Consequently, the primary CAI failure mode for stitched specimens is fracture-strength failure.
(4) Unimpacted specimens exhibit higher compressive strength than damaged ones, primarily because impact damage induces crack initiation and propagation, leading to interlaminar debonding. The larger the damaged area, the lower the residual strength. Therefore, the stitching process helps reduce the delamination area, enhancing the residual compressive strength of the laminate. Higher stitching density correlates with greater residual compressive strength. At 20 J, the strength of specimens with three different stitch densities increased by 6.52%, 17.71%, and 27.48%, respectively.

## Figures and Tables

**Table 1:** The properties of the resin.

Brand Type	R668
Appearance	Colorless transparent liquid
Viscosity (mPass@25 °C)	1100–1200
Density (g/cm^3^)	1.1–1.2
Flash Point (°C)	185
Storage temperature (°C)	2–40

**Table 2:** Test specimen information.

Specimen Number	Specimen Type	Stitch Density (mm × mm)	Specimen Thickness (mm)	Impact Energy (J)
UL	Unstitched	/	2.4	0/10/15/20
SL1010	Stitched	10 × 10	2.4	
SL1015	Stitched	10 × 15	2.4	
SL1515	Stitched	15 × 15	2.4	
UL-T	Unstitched	/	4.8	
SL1010-T	Stitched	10 × 10	4.8	
SL1015-T	Stitched	10 × 15	4.8	
SL1515-T	Stitched	15 × 15	4.8	

## Data Availability

The original contributions presented in this study are included in the article. Further inquiries can be directed to the corresponding authors.
